# Postnatal foot length in the estimation of gestational age in relation to intrauterine growth pattern among Nigerian neonates

**DOI:** 10.4314/gmj.v57i3.11

**Published:** 2023-09

**Authors:** Opeyemi T Kuponiyi, Tinuade A Ogunlesi

**Affiliations:** 1 Department of Paediatrics, Olabisi Onabanjo University Teaching Hospital, Sagamu, Ogun State, Nigeria; 2 Department of Paediatrics, Olabisi Onabanjo University, Sagamu, Ogun State, Nigeria

**Keywords:** Gestational Age, Foot length, Intra-uterine Growth Pattern, Prematurity

## Abstract

**Objectives:**

To determine the relationship between postnatal foot lengths and estimated gestational age (EGA) in relation to intrauterine growth patterns determined at birth among Nigerian neonates.

**Design:**

Hospital-based, cross-sectional.

**Setting:**

Olabisi Onabanjo University Teaching Hospital, Sagamu, Nigeria.

**Participants:**

260 neonates with EGA 30- 42 weeks within 48 hours of life.

**Interventions:**

Postnatal foot lengths (FL) were measured with Vernier digital calliper in millimetres. The intra-uterine growth pattern was determined using the Lubchenco chart. Pearson correlation and regression analysis tests were performed.

**Main outcome measures:**

Postnatal foot length in relation to Intra-Uterine Growth Pattern

**Results:**

The mean postnatal FL had a strong positive correlation with the EGA from 30 through 42 weeks (r = 0.855, p < 0.001). The overall mean foot length for preterm neonates was 65.44 (6.92) mm, while that of term neonates was 77.92 (4.24) mm. The linear regression equation was generated as: EGA = 9.43 + (0.37 × FL), p < 0.001. The EGA as measured by FL had the highest positive correlation with Small for Gestational Age (SGA) intra-uterine-growth pattern, followed by Appropriate for Gestational Age (AGA) and least by Large for Gestational Age (LGA) respectively (r = 0.936> 0.861 > 0.666).

**Conclusion:**

The postnatal foot length correlated well with estimated gestational age, and the correlation was best among SGA infants.

**Funding:**

None declared

## Introduction

Gestational age, also known as menstrual age, refers to the age of the foetus calculated from the first day of the mother's last menstrual period.^1^ The gestational age of any foetus indicates the degree of maturity of such foetus and can prognosticate the survival of the neonate. Therefore, the gestational age must be accurately determined at all levels of health care delivery to assess foetal growth, risk designation, management, prognostication and follow-up care.^2^ In standard practice, the New Ballard Score is used to estimate babies' gestational age at birth.^3^ This scoring system assesses a range of six physical and six neurological parameters in determining the approximate gestational age to the nearest two weeks, although the most ideal to use is the gestational age extrapolated from an early cyesis ultrasound scan.

About 50% of deliveries in sub-Saharan Africa occur at home, and neonates are often presented first at these lower tiers of healthcare delivery where these scoring systems are not used due to lack of proficiency by the healthcare givers.^4^,^5^,^6^ These at-risk, vulnerable home-delivered ones are missed, worsening Africa's neonatal morbidity and mortality indices. Low levels of maternal education, poor recall of LMP by pregnant women and variation in the timing of ovulation contribute to discrepancies in EGA determination by LMP.^7^ Late antenatal booking and poor utilisation of the obstetric ultrasound scan service in the early trimester for gestational age determination due to ignorance, poverty, low accessibility and unavailability of ultrasound scan (USS) services reduce the chances of accurate gestational age determination using early cyesis ultrasound scan.^6^, ^7^

The World Health Organisation (WHO)^8^ approved using neonatal anthropometry as surrogates or proxy for weight and gestational age estimation.

The intra-uterine life is characterised by rapid somatic growth manifesting as increasing foetal length and progressive increase in foot length, all in relation to increasing gestational age, as previously reported by Wong.^9^ The foot is a readily accessible body part even in very sick and premature babies; hence may be measured. The plausible relationship between postnatal FL and the gestational age of neonates may be adapted for use at the most basic levels of health care using basic anthropometry techniques. Suppose a relationship is established between the postnatal FL and EGA. In that case, the management of ill neonates can be more rapidly instituted as clinical methods of EGA determination are time-consuming and require greater skill than postnatal FL measurements. In settings where a decision needs to be made, with respect to whether a baby is preterm or term or, if preterm, how immature, the foot length may be useful. For example, preterm babies are not allowed to feed by direct suckling up until 34 weeks because of the disconnect between swallowing and sucking before 34 weeks, Daga *et al*,^10^ showed that a postnatal foot length of 6.5 cm is equivalent to 34 weeks of gestation, hence, may be used to guide that crucial decision on preterm infant feeding. This is supported by more recent studies which have demonstrated strong correlations between estimated gestational age and postnatal foot length.^11^,^12^

Foetal growth is usually assessed by comparison of the weight, body length and OFC at birth with standards that define reference ranges at a spectrum of gestational ages, called the intrauterine growth charts. Lubchencho *et al*^13^ developed the first intra-uterine growth charts involving only Caucasian neonates, and many more growth charts were developed in different parts of the world. Using the Lubchenco curves, neonates spanning 24-42 weeks of gestation can be classified based on the intra-uterine growth status into groups: Small- for- Gestational Age (SGA), Appropriate-for-Gestational Age (AGA) and Large-for-Gestational Age (LGA).^14^ It is important to consider the pattern of intra-uterine growth into consideration while using the postnatal foot length as a proxy for gestational age since foot length, as a form of somatic growth, may theoretically vary with other anthropometric parameters, depending on intra-uterine nutritional and growth status.

There have been studies on postnatal foot length in estimating gestational age, mostly outside Africa. These studies reported a good correlation between foot length and gestational age.^15^-^17^ However, these studies are sparse in West Africa, and only very few addressed the possible role of differing intra-uterine growth patterns.

Therefore, the study's objective was to determine the relationship between postnatal foot lengths and estimated gestational age in relation to intra-uterine growth patterns among Nigerian neonates.

## Methods

This study was conducted at the Olabisi Onabanjo University Teaching Hospital, Sagamu, a semi-urban part of Ogun State, South-West, Nigeria. The hospital offers specialised obstetric and paediatric services limited to a Level II perinatal/ neonatal care services. The hospital has approximately 677 deliveries and 452 neonatal admissions annually, with referrals from all tiers of healthcare in neighbouring states.

### Study design

The study was a hospital-based, cross-sectional survey between December 2017 and December 2019.

### Study population

Participants were consecutive singleton neonates delivered or presented at the hospital following referral. The inclusion criteria included age between 0 and 48 hours, estimated gestational age from 30 to 42 completed weeks determined using the New Ballard Score (NBS) method and known birth weight. Neonates with obvious gross congenital malformations and those with lower limb deformities (including but not limited to Talipes equinovarus, Talipes calcaneovalgus and arthrogryposis) were excluded.

### Sample size

The minimum sample size was determined using the formula for determining a population mean with precision:^18^ N = Zβ^2^σ^2^/ε^2^ where n is the minimum sample size, Zα is Standard normal co-efficient = 1.96 at 95% Confidence Interval, SD(σ) is the standard deviation obtained from a previous study,^2^ and ε is the margin of error (5%) above the mean obtained from a reference study.^2^ A minimum of 20 neonates was calculated for each gestational age (30 – 42 weeks) with 260 neonates.

### Ethical considerations

Ethical approval was obtained from the Health Research Ethics Committee of the hospital with approval number OOUTH/HREC/111/2017, and written informed consent was obtained from the mothers/caregivers at the point of enrolment into the study.

### Data collection

The age (in hours), sex, and the NBS-derived EGA were recorded using the research proforma. Only one author (KOT) measured EGA and foot length. She had earlier received in-house training on how to take the measurements using a calliper.

Two foot length measurements (FL) were made using the battery-operated Vernier Digital Calliper (Pittsburgh 6in Digital Calliper, Harbor Freight, USA). The average of the two-foot lengths was recorded for each infant. For reliability, the batteries were replaced after every tenth use to avoid fluctuations in the tool's capacity. Each neonate's foot length (in millimetres) was measured on the sole of both feet, between the heel and the tip of the second toe of each foot, without exerting pressure. The tip of the second toe was adopted, being the longest of the toes anatomically. A research assistant stabilised The neonate's ankle (leg and foot at 900). The Vernier Calliper displayed values to one decimal place with a margin of error of 1/20mm or 0.05mm. 19 The average of the two measurements recorded for both feet using the calliper was recorded for each neonate. The intra-class reliability coefficient was 0.991 (with p<0.0001).

The EGA was used to determine intra-uterine growth patterns on the Lubchenco charts,13 validated among Nigerian babies. The intra-uterine growth pattern was classified using the birth weight into Small-for-Gestational Age (SGA) (plotting below the 10^th^ centile for the gestational age), Appropriate-for-Gestational Age (AGA) (plotting between the 10^th^ and 90^th^ centiles for the gestational age) or Large-for-Gestational Age (LGA) (plotting above the 90^th^ centile for the gestational age). 14, 20 Infants with EGA less than the 37^th^ completed weeks were classified as preterm, while those with EGA of at least 37^th^ completed weeks were classified as term.

### Data analysis

The Statistical Package for Social Sciences (SPSS) software version 20.0 was used for data analysis. Continuous variables were described as mean and standard deviations, while categorical data were reported as proportions and percentages. The mean values and standard deviations were compared using Student's t-test or One-Way ANOVA test, while proportions were compared using the Chi-Square test and Odds ratio. The relationships between continuous variables (FL was the predictor variable while EGA was the outcome) were determined using Pearson correlation or Linear regression tests. Statistical significance was determined by p-values less than 0.05 or 95% Confidence Interval (CI) excluding unity.

## Results

A total of 260 neonates, comprising 140 (53.8%) preterm and 120 (46.2%) term neonates, were studied. The mean (SD) age at enrolment was 24.64 (15.38) hours. The neonates comprised 141 (54.2 %) females and 119 (45.8%) males. An appropriate-for-gestational-age subgroup of babies accounted for 85% (221/260) of the study population, followed by the SGA subgroup (9.2%, 24/260), as shown in Table 1.

**Table 1:** Demographic characteristics of neonates and their mothers and maternal obstetric factors

Variable		Frequency	Percentage
**Age (Hours)**	1-12	66	25.4
	13-24	78	30.0
	25-36	45	17.3
	36-48	71	27.3
**Sex**	Female	141	54.2
	Male	119	45.8
**IUGP**	*AGA	221	85.0
	*SGA	24	9.2
	*LGA	15	5.8
**Maturity at Birth**	Term	120	46.2
	Preterm	140	53.8

*IUGP- Intra-Uterine Growth Pattern *SGA- Small for Gestational Age *AGA- Appropriate for Gestational Age; *LGA- Large for Gestational Age. These sub-groups were determined by plotting the birthweights and estimated gestational age (EGA) on the Lubchenco chart.

The mean (SD) foot length increased with increasing gestational age, as shown in Table 2. The mean foot length for preterm neonates was 65.44 (6.92) mm, while that of term neonates was 77.9 2 (4.24) mm.

**Table 2:** Mean foot length across gestational ages

EGA	N	Mean (mm)	SD
**30**	20	58.86	2.69
**31**	20	59.99	4.47
**32**	20	60.62	4.37
**33**	20	64.84	3.24
**34**	20	68.29	5.17
**35**	20	70.50	5.55
**36**	20	74.67	3.26
**37**	20	76.25	4.32
**38**	20	77.06	3.54
**39**	20	76.24	3.48
**40**	20	78.37	4.43
**41**	21	78.62	3.82
**42**	19	81.37	4.19
**Total**	260	71.21	8.59

**EGA – Estimated Gestational Age; SD – Standard deviation.**

Of the preterm neonates, 124 (88.6%) were AGA, 15 (10.7%) were SGA, and one neonate was LGA. The mean differences in foot length across the IUGP were statistically significant for the preterm neonates. Of the term neonates, 97 (80.8%) were AGA, eight (6.7%) were SGA, and 15 (12.5%) were LGA. The mean differences of foot length across the EGA per type of Intra-Uterine Growth Pattern (IUGP) were statistically significant except at 38 and 42 weeks as shown in Table 3. In preterm babies, the AGA group had significantly longer mean foot lengths than the SGA group. LGA groups, particularly among term babies, had longer mean foot lengths compared to the AGA group, with the lowest mean foot lengths observed among SGA groups.

**Table 3:** Mean foot length across patterns of intra-uterine growth in each EGA

EGA (weeks)	AGA	SGA	LGA	Statistics	p-value	Mean difference	95%,CI
**30**	n=20	n=0	n=0				
	58.86 (2.64)			NC			
**31**	n=16	n=4	n=0	t = 4.21	0.001	7.68	3.68,11.51
	61.53 (3.43)	53.85 (2.15)					
**32**	n=16	n=4	n=0				
	61.76 (4.06)	56.07 (1.99)		t = 2.68	0.015	5.68	1.22,10.14
**33**	n=20	n=0	n=0				
	64.84 (3.24)			NC			
**34**	n=18	n=2	n=0				
	69.10 (4.77)	60.95 (0.63)		t = 2.35	0.03	8.15	8.79,15.43
**35**	n=16	n=4	n=0				
	72.27 (4.34)	63.40 (4.17)		t = 3.68	0.002	8.87	3.0, 13.94
**36**	n=18	n=1	n=1				
	74.67 (2.87)	67.70	78.20	F = 3.73	0.045	NC	NC

**Figure 1:**
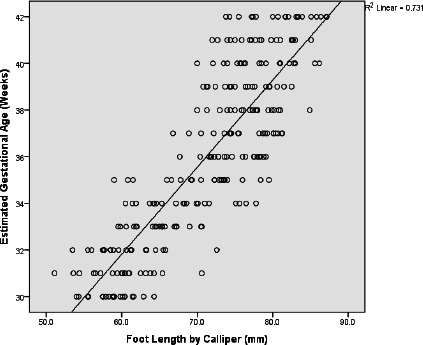
Scatterplot of the mean foot length across gestational ages

Figure 1 shows that the overall foot length had a strongly positive correlation coefficient (r = 0.855) with EGA with a linear regression coefficient, R^2^ = 0.731. The linear regression equation was generated as: EGA = 9.43 + (0.37 × FL), p < 0.001.

**Figure 2:**
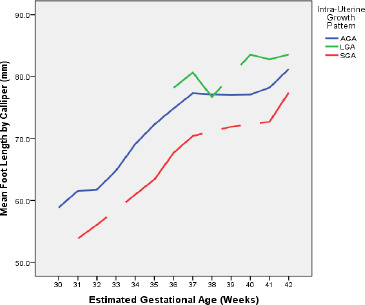
Comparison of mean FL across patterns of intra-uterine growth in each EGA

Figure 2 shows the intrauterine growth curves of the AGA, SGA and LGA in the neonates. The growth curves of these three IUGPs followed a similar trend. The AGA curve showed a sharp rising slope from the early gestation till about the 37^th^ week and a slowing of the gradient thereafter till term. The SGA curve followed a similar pattern. However, the LGA curve could not be fully explored at the early gestational ages (30-35 weeks) as there were no subjects with LGA. The LGA curve showed a similar pattern to the AGA and SGA curves within term gestation, save a dip at about 38 weeks.

Mean postnatal foot length strongly correlated with EGA in SGA and AGA neonates. The SGA group had the highest correlation (r = 0.936) and highest correlation coefficient (87.6%), followed by the AGA group. The regression coefficient represents the proportion of the model that was truly due to a relationship between the postnatal foot length and EGA. The correlation values are shown in Table 4.

**Table 4:** Correlation between foot length and EGA per intra-uterine growth pattern

IUGP	r	R^2^	P
**AGA**	0.861	0.741	<0.001
**SGA**	0.936	0.876	<0.001
**LGA**	0.666	0.443	0.07

*IUGP= Intra-uterine growth pattern, r = Correlation coefficient, R^2^ = Regression co-efficient; p = level of significance, SGA= small-for-gestational-age; LGA=Large-for-gestational-age; AGA= Appropriate-for-gestational-age

## Discussion

The mean postnatal foot length of the neonates increased with increasing gestational age. The mean FL (71.21±8.59mm) in this study is similar to the mean postnatal FL observed by Dagnew *et al*^21^ in Ethiopia (74.1 + 0.67mm) and Srinivasa *et al*^22^ in India (75.8 ± 0.44 mm) with sample sizes of 205 and 500 neonates respectively, consisting of preterm and term babies. Also, the mean postnatal FL was higher in term newborns than in the preterms in this study. This affirms the position that a positive linear relationship exists between the postnatal FL and the EGA, stressing the increasing somatic growth as the gestational age increases. The mean postnatal FL of the neonates were not stated in a South Africa study by Van *et al* 2 but a slightly reduced range (59mm-75mm) could be deduced from the report, possibly due to the small sample size (n=85), comprising of only AGA preterm and term neonates. Compared to the above studies, the mean postnatal FL suggests that the measurement of the postnatal foot length in this study gave a reliable assessment of the variable.

The strong correlation between the mean postnatal FL and the EGA, as displayed by the scatterplot of these two variables in the study, supports the increase in somatic growth as gestational age increases, including increase in the size of limb structures. Similar findings of positive, strong correlation and statistical significance have been documented by earlier research in parts of Africa and Asia despite variations in sample sizes, thus affirming the relationship.^2^,^21^

The NBS method for estimating the EGA was adopted in the study by Van *et al*^2^ in South Africa, Dagnew *et al*^21^ in Ethiopia and Srivastava *et al*^23^ in India as in this study and they reported a similarly high correlation of postnatal FL with the EGA. However, a similar study with a relatively large sample size (424) in Ethiopia by Tiruneh *et al*^24^, in which the LMP was utilised for the EGA, reported a poor correlation coefficient (r = 0.143) of the postnatal FL with the EGA for no known reasons.

The regression equation generated showed that the EGA increased by 3.7 weeks if the postnatal FL increased by 10mm. For a hypothetical postnatal FL of 60mm, the calculated EGA will be 31 completed weeks, which is very close to the mean value of postnatal FL in the present study (59.99mm). The R^2^ value infers that the regression equation correctly predicted the gestational age in 73.1% of cases, strongly influencing the prediction of gestational ages in weeks. The import of this cannot be overemphasised as the generated equation would allow for timely estimation of the EGA in clinical practice, with no requirement for clinical expertise as needed in using the NBS method.

A strong correlation coefficient was reported by another study^21^ in another part of Africa in consonance with the present study. Extension outside the 72-hour period for the use of the NBS method of EGA may have an influence on lowering the degree of correlation, as observed in the study by Fawzia *et al**25* in India. The foot length also had a positive, strong linear relationship with the EGA along the intra-uterine growth patterns. This compared to the findings by Srivastava *et al*,^23^ Rakkappan *et al*,^26^ Tenali *et al*^27^ and Gavhane *et al*^28^ in parts of India where the foot length positively correlated with gestational age in SGA and AGA groups. This is due to a characteristic pattern of normal growth of the foetal foot with no significant variation in its growth and that the foetal foot is least affected by intrauterine growth restriction and extremes of growth abnormalities.^29^ This makes the foot length a good predictor of gestational age despite intra-uterine nutrition patterns. Also, the correlation was better with foot length across the EGA due to less growth variation in the SGA group. This observation may be clinically indispensable since about 7.2% and 77% % of births are SGA and AGA, respectively in Nigeria.^30^ Also, it is important that the moderate correlation of the postnatal FL with the LGA group of neonates be interpreted with caution due to the low representation of the group in the study analysis. The low representation of the LGA group in population-based studies in neonates has been documented particularly in low-resource settings.^26^, ^30^ In agreement with this study, Tenali *et al**27* in India also reported a positive lower correlation, which was also not statistically significant.

The line graphs of the AGA and SGA babies followed a similar pattern from the preterm to the term period whilst the LGA subgroup was the least represented (5.8%) as there was a lack of data in the LGA group, particularly among the preterm neonates. The mean differences in the foot length amongst these three intrauterine growth patterns were mostly statistically significant as each pattern is a distinct foetal nutrition/ growth entity. A slowing in the gradient of the mean foot length across the three patterns from about 37 weeks EGA corresponds with the commencement of the term period with a reduction in growth velocity possibly resulting from waning placental sufficiency. A dip in the line graph of the LGA group at about 38 weeks may be an exaggeration of the growth restriction for unknown reasons.

This study demonstrated that there is a correlation between the postnatal foot length and the EGA, and has the potential to help neonatal caregivers make informed care decisions in resource-limited settings.

A limitation of the study is that the study only enrolled neonates of gestational ages 30 weeks to 42 weeks due to the low- admission rate of extremely preterm and post term neonates at the hospital. Therefore, the earlier and later gestational ages were not studied.

The hospital setting of the study may be a limitation since the study was aimed at addressing a problem that occurs outside the hospital. However, this study has established a methodology which can be deployed in subsequent community-based studies. In another arm of the study, a flexible but inelastic plastic tape rule was compared with a calliper in the measurement of postnatal foot length, given the challenges associated with using a calliper in the community (In press). The use of NBS in estimating gestational age is also acknowledged as a limitation since early cyesis ultrasound determined gestational age, which should have been the best option, was not possible in many of the mothers, given their education and socioeconomic background.

## Conclusion

The postnatal foot length had a strong positive linear association with the estimated gestational age from 30 weeks through 42 weeks, and the postnatal foot length had the highest positive correlation with the estimated gestational age among SGA neonates, followed by the AGA subset and least correlation with the LGA neonates across the EGA. The normative data of the study's gestational age-specific postnatal foot length may be used to estimate gestational age where the skill for NBS is unavailable. The linear regression equation derived from this study can also be used to generate EGA from measured FL.
